# Investigation into Brazilian Palygorskite for Its Potential Use as Pharmaceutical Excipient: Perspectives and Applications

**DOI:** 10.3390/ma16144962

**Published:** 2023-07-12

**Authors:** Lyghia Maria Araújo Meirelles, Raquel de Melo Barbosa, Rita Sanchez-Espejo, Fátima García-Villén, Luana Perioli, César Viseras, Tulio Flavio Accioly de Lima e Moura, Fernanda Nervo Raffin

**Affiliations:** 1 Department of Pharmacy, Federal University of Rio Grande do Norte, R. Gen. Gustavo Cordeiro de Faria, s/n—Petrópolis, Natal 59012-570, Brazil; lyghiamaria@unifsa.com.br (L.M.A.M.); tulio.moura@ufrn.br (T.F.A.d.L.e.M.); fernanda.raffin@ufrn.br (F.N.R.); 2 Department of Pharmacy and Pharmaceutical Technology, School of Pharmacy, University of Granada, Campus of Cartuja s/n, 18071 Granada, Spain; ritamsanchez@ugr.es (R.S.-E.); fgarvillen@ugr.es (F.G.-V.); cviseras@ugr.es (C.V.); 3 NanoBioCel Group, Laboratory of Pharmaceutics, School of Pharmacy, University of the Basque Country UPV/EHU, 01006 Vitoria-Gasteiz, Spain; 4 Department of Pharmaceutic Science, University of Perugia, 06123 Perugia, Italy; luana.perioli@unipg.it; 5 Andalusian Institute of Earth Sciences, CSIC-University of Granada, Av. de Las Palmeras 4, 18100 Armilla, Spain

**Keywords:** palygorskite, excipients, pharmaceutical applications, clay mineral

## Abstract

Palygorskite is an aluminum and magnesium silicate characterized by its fibrous morphology, providing it with great versatility in industrial applications, including pharmaceuticals. Although most of the reserves are in the United States, in recent years occurrences of commercially exploited deposits in Brazil have been recorded, mainly in the country’s northeast region. This has motivated this study, which analyzes raw Brazilian palygorskite compared to a commercial sample (Pharmasorb^®^ colloidal) to demonstrate its pharmaceutical potential. The chemical and mineral composition of the samples were evaluated for surface properties, granulometry, morphology, crystallography, thermal analysis, and spectroscopy. Raw palygorskite presented 67% purity, against 74% for Pharmasorb^®^ colloidal. The percentage purity relates to the presence of contaminants, mainly carbonates and quartz (harmless under conventional conditions of pharmaceutical use). Furthermore, it was possible to confirm the chemical composition of these phyllosilicates, formed primarily of silicon, aluminum, and magnesium oxides. The crystallographic and spectroscopic profiles were consistent in both samples, showing characteristic peaks for palygorskite (2θ = 8.3°) and bands attributed to fibrous phyllosilicates below 1200 cm^−1^, respectively. The thermal analysis allowed the identification of the main events of palygorskite, with slight differences between the evaluated samples: loss of water adsorbed onto the surface (~85 °C), removal of water contained in the channels (~200 °C), coordinated water loss (~475 °C), and, finally, the dehydroxylation (>620 °C). The physicochemical characteristics of raw palygorskite align with pharmacopeial specifications, exhibiting a high specific surface area (122 m^2^/g), moderately negative charge (−13.1 mV), and compliance with the required limits for heavy metals and arsenic. These favorable technical attributes indicate promising prospects for its use as a pharmaceutical ingredient in the production of medicines and cosmetics.

## 1. Introduction

Clays are naturally occurring materials composed mainly of finely divided minerals, which have plastic behavior in the presence of water and harden when dry [1]. The most relevant properties of these materials refer to the nanometric size of the structural units and the presence of mesopores and micropores, which give them a high contact surface, a variable surface charge, adsorptive capacity, and hydrophilicity [2,3]. Such characteristics make these inorganic materials attractive for some pharmaceutical applications. Although official textbooks have recognized some clay minerals as pharmaceutical excipients, these materials have been used as drug carriers for many decades [4,5,6,7]. These nanomaterials have been used for various purposes, such as increasing drug solubility, improving stability, or modifying drug release profiles [8,9,10,11]. Among the groups of phyllosilicates described in pharmacopoeias, and therefore are apt for use in pharmaceutical products, kaolin, talc, montmorillonite, bentonite, palygorskite, and sepiolite stand out [12].

Palygorskite, most commonly known as attapulgite in pharmaceutical texts, is an aluminum and magnesium silicate characterized by its tunnels and rectangular channels, of which their restricted dimensions allow for the insertion of small molecules and cations [13,14]. The highly adsorptive properties of palygorskite are due to its moderately negative surface charge, intermediate cation-exchange capacity, and high specific area [13]. The main deposits of palygorskite can be found in the United States, but there are some commercially exploitable deposits in Brazil, more particularly in the northeastern region of the country [15]. Given its availability, accessibility at a low cost, and the versatility of applications for which it can be used, palygorskite attracts great industrial interest. In the pharmaceutical field, it can be used as a diluent, adsorbent, and disintegrant in solid pharmaceutical forms; colloidal palygorskite is able to form gels and stabilize emulsions, as well as treat diarrhea [16,17].

The fact that palygorskite is an inorganic material that is biocompatible and capable of composing pharmaceutical preparations reinforces its use in the development of drug delivery systems [11,18,19,20,21,22,23]. Therefore, this study characterizes a palygorskite extracted from a Brazilian reserve in order to demonstrate its potential pharmaceutical use by performing comparative analyses. To do so, a palygorskite sample, commercially available and already authorized as a pharmaceutical excipient (Pharmasorb^®^ colloidal), was used as a reference.

## 2. Materials and Methods

### 2.1. Materials

The palygorskite sample, provided by Ummio Indústria de Minérios Ltd.a (São Pedro, Piauí, Brazil), was extracted in the region of Guadalupe, latitude 06°47′13″ south and longitude 43°34′09″ west (Piauí, Brazil). This will be characterized as ‘raw palygorskite’ in this study. The sample was then characterized and compared to pharmaceutical-grade palygorskite, Pharmasorb^®^ colloidal (BASF, Limburgerhof, Germany), which was used as received. Methylene blue extra-pure grade was purchased from Sigma Aldrich, St. Louis, MO, USA. All other chemicals and solvents were of analytical grade. Deionized water (18.2 MW cm) was obtained from a Waters ultrapure water system.

### 2.2. Methods

#### 2.2.1. Pretreatment of Raw Palygorskite

An aqueous dispersion of palygorskite was prepared (1:10, *w*/*v*), which allowed the denser suspended solids to settle, and the supernatant was collected. The suspension containing the finest particles was filtered on filter paper, and the material retained on the filter was then dried in an oven at 60 °C for 24 h. The dried product was sieved through a 200-mesh sieve, obtaining a final yield of approximately 76%. This procedure was performed before starting the analysis to remove any coarse sand and other foreign agents from the sample.

#### 2.2.2. Chemical and Mineralogical Composition

The chemical composition of the major elements present in clay minerals was determined by X-ray fluorescence (XRF), using a Bruker S4 Pioneer spectrometer (Karlsruhe, Germany), equipped with an Rh anode X-ray tube, voltage of 60 kV, and current of 150 mA. Powder X-ray diffraction (XRD) of the samples was performed using a Shimadzu diffractometer, model XDR-6000 (Kyoto, Japan), with Cu kα radiation, 30 kV, 30 mA, 5–70° (2θ) scan range, in Rietveld mode. Mineralogical composition was determined by the combination of data obtained in the XRF and XRD analyses, according to the method of López-Galindo et al. [24].

#### 2.2.3. Inductively Coupled Plasma Optical Emission Spectroscopy Analysis (ICP-OES) for Heavy Metals and Arsenic

ICP-OES was employed as the analytical technique to determine the presence of heavy metals and arsenic in the samples of raw Palygorskite and Pharmasorb^®^ colloidal. Sample preparations were carried out using the Cem Corporation Mars 5 microwave sample preparation digester (Matthews, NC, USA). Equal masses of 0.50 g were weighed into the digestion vessel, and 10 mL of HNO_3_ (Êxodo Científica, São Paulo, Brazil) was added. The mixture was gently agitated, allowing for a 15 min waiting period before sealing the vessel. Subsequently, the vessel was placed in the equipment following the methodology for a single-stage mineral oil sample at a temperature of 200 °C, with a ramp and hold time of 15 min, a pressure of 800 psi, and a power range of 900–1050 W. After partial digestion, the samples were filtered and diluted with high-purity deionized water. The sample preparation method used was EPA 3051A from the Environmental Protection Agency (United States Environmental Protection Agency, 1996), as recommended by SDA Instruction Normative No. 24 of 2007 [25].

#### 2.2.4. Methylene Blue Adsorption (MBA)

MBA measurements were conducted following Cerezo and co-authors (2001), with some modifications [26]. Ten milliliters of silicate/water dispersion at 10% (*w*/*v*) was agitated for 15 min at 10,000 rpm in the presence of an 80 mL solution of 0.1% (*w*/*v*) methylene blue in water. Subsequently, the resulting product underwent centrifugation, and 5 mL of the resulting solution was diluted with water up to 500 mL. The absorbance of the diluted solution at 625 nm was measured using a UV–visible spectrometer (Evolution 300), Thermo Scientific, Waltham, MA, USA. MBA was calculated using the calibration curve y = 0.2371x − 0.0218 (r^2^ = 0.999), which was obtained for methylene blue from solutions with known concentrations ranging from 0.5 to 3.0 μg/mL.

#### 2.2.5. Loss on Drying

The moisture content of the clays was determined from the drying of 1 g of the samples, under 105 °C, until constant weight in an oven (Q317M-22, Quimis, São Paulo, Brazil) [27].

#### 2.2.6. Acidity and Alkalinity

A 5% (*w*/*v*) aqueous dispersion was prepared in carbon dioxide-free water, after magnetic stirring for 5 min. The pH was measured using a pHmeter (Q400AS, Quimis, São Paulo, Brazil). The electrode was previously calibrated using buffer solutions of pH 4, 7, and 10 [27].

#### 2.2.7. Surface Properties and Particle Size Analysis

Specific area was determined using the BET method (Brunauer–Emmett–Teller) through adsorption–desorption isotherms of N_2_ in a specific area and pore analyzer Beckman Coulter, model SA 3100 (Brea, CA, USA). Before measuring, the samples were degassed under vacuum at constant temperature (120 °C) for two hours. Particle size analysis was performed in a Cilas laser granulometer, model 1090 (Orléans, France). Each sample was deposited in the feeder device, in dry mode, under vibration at a frequency of 55 Hz. Powders were propelled into the analyzer by compressed air under 2500 mb. The particle size distribution is determined by the diameters at 10%, 50%, and 90% of the cumulative volume of distribution, respectively, d10, d50, and d90 (µm). Span, calculated from Equation (1), is an index that determines the range of distribution of fractions with different particle sizes.

(1) $$Span=\frac{d_{90}-d_{10}}{d_{50}}$$

Zeta potential (ζ) was determined at 25 °C using Zetasizer Nano ZS Malvern (Worcestershire, UK). The samples were suspended in water at a concentration of 0.1% (*w*/*v*) and the values obtained corresponded to the average of twenty runs, which were performed in triplicate for each sample.

#### 2.2.8. Fourier Transform Infrared Spectroscopy

Fourier Transform Infrared (FTIR) spectra were recorded on a Shimadzu FTIR spectrometer, IR Prestige-21 model (Tokyo, Japan) equipped, with an attenuated total reflectance (ATR) accessory. Measurements were performed at an interval of 700–4000 cm^−1^, at a resolution of 4 cm^−1^.

#### 2.2.9. Thermal Analysis

Thermogravimetric (TG) and differential thermal analyses (DTA) were performed using a Shimadzu thermobalance, model DTG-60 (Tokyo, Japan), in the range of 30–800 °C. A precision scale was used to measure the 5.0 ± 0.5 mg of sample used, contained in an alumina crucible, and heated at 10 °C/min in a nitrogen atmosphere with a flow of 50 mL/min.

#### 2.2.10. Transmission Electron Microscopy Analysis

Palygorskite samples were deposited on a Cu grid to determine their morphology in a Philips CM20 High Resolution Transmission Electron Microscope (HRTEM) operated at 200 kV (Dresden, Germany).

## 3. Results

### 3.1. Chemical and Mineralogical Composition

Data from the chemical analysis of the studied samples are shown. By evaluating the chemical composition, Si, Mg, and Al are observed as the major elements in both specimens, demonstrating that it is a magnesium and aluminum silicate (Table 1).

Clay mineral samples from natural sources commonly have varying compositions, depending on the location they were extracted from, and their mineralogical classification. Some of the minerals or organic substances associated with phyllosilicates are quartz, feldspar, carbonates, sulphates, iron, and aluminum oxides, among others [12].

**Figure 1:**
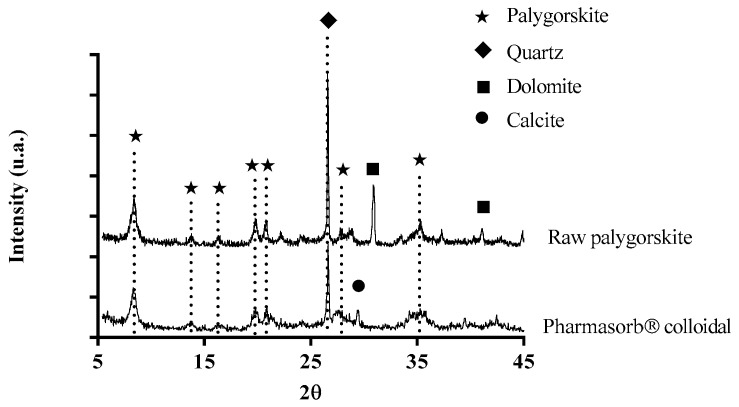
X-ray diffractograms of raw palygorskite and Pharmasorb^®^ colloidal showing the main mineral components of the samples.

The composition of an ideal cell of palygorskite corresponds to (Mg, Al)_5_Si_8_O_20_(OH)_2_(OH_2_)_4_·4H_2_O, which justifies its characterization as a magnesium and aluminum silicate [28]. However, it is rare to find samples of palygorskite with a purity higher than 75%. Quartz (SiO_2_), calcite (CaCO_3_), and dolomite CaMg (CO_3_)_2_ are the minerals most frequently associated with this phyllosilicate [12]. The same crystalline phases identified in the mineralogical analysis of raw palygorskite had already been found in similar samples analyzed by our group [20], as well as in a previous characterization of Pharmasorb^®^ colloidal [29]. Thus, the highest value of SiO_2_ in relation to the other oxides is related to the main components of the samples (palygorskite and quartz) (Table 1). The main recommendations for the use of palygorskite, as an excipient in oral and topical pharmaceutical formulations, do not pose a risk of exposure to high levels of quartz through these routes [30]. Furthermore, the American Pharmacopoeia does not present any restriction on the content of these particles, and recent publications reinforce that the use of pharmaceutical products containing quartz does not pose a relevant risk when administered orally and topically [31,32]. The highest relative composition of SiO_2_ in Pharmasorb^®^ colloidal is associated with the degree of purity of this sample, which is higher than in raw palygorskite, corroborated by the diffraction analysis (Figure 1). Baltar and collaborators (2009) reported that palygorskite samples obtained from the same deposit as raw palygorskite had equivalent quartz content (15.5–18.0%) [33]. 

Crystallographic analysis of the samples confirmed the results obtained by XRF, observing the characteristic pattern of palygorskite in the spectrum of Figure 1, with a more intense reflection at 8.3°, followed by other smaller reflections, also corresponding to palygorskite, at 2θ = 13.8°, 16.3°, 19.8°, 20.8°, 27.7°, and 35.3°. The obtained diffraction patterns are in agreement with the literature [20,34,35]. A slightly higher proportion of calcium magnesium oxide in raw palygorskite was identified when compared to Pharmasorb^®^ colloidal. This finding can be attributed to the presence of associated minerals such as dolomite (Figure 1). The formation of palygorskite is favorable in arid and semiarid regions in the presence of carbonates and alkaline medium, rich in magnesium, with the crystallization of dolomite [36,37]. Pharmasorb^®^ colloidal also has a small amount of calcite, the most common type of calcium carbonate, which was also identified in a previous analysis of this sample [18].

In addition to the reflections of phyllosilicate, the impurities identified in the mineralogical analysis, such as quartz and carbonates, can be identified in the diffractogram. Dolomite was identified in raw palygorskite at 2θ = 30.9° and 41.1°, while calcite (2θ = 29.4°) can be seen in the diffractogram of Pharmasorb^®^ colloidal. These data indicate that even the pharmaceutical-grade palygorskite has a certain level of impurities [38]. 

Iron detected in the FRX analysis is a common element in palygorskite samples, since the Fe^3+^ cation is present in the octahedral sheet. The percentage of iron oxide between 3 and 5% is consistent with the mean values reported by other authors [20,39,40]. 

Some of the transition metals identified in the composition of phyllosilicates, such as iron, manganese, and copper (Table 1), are of pharmaceutical interest due to their potential catalytic properties in oxidative reactions of drugs, which may have a negative impact on the stability of drugs that are susceptible to oxidation [41].

The proportion of manganese oxide in raw palygorskite was 0.27% and is in agreement with samples from the same region (0.21%) as found by Pereira et al. [40]. However, it has a value approximately five times higher than that of commercial palygorskite. These variations are related to the mechanism by which the clay mineral was formed and its origin [42,43]. 

The combination of XRF and XRD results demonstrate the mineralogical composition of raw palygorskite as follows: palygorskite (67%), dolomite (15%), and quartz (13%). Pharmasorb^®^ colloidal, in turn, is composed of palygorskite (74%), quartz (10%), and calcite (5%). The diffractograms of raw palygorskite and Pharmasorb^®^ colloidal are shown in Figure 1 and show the crystalline profile of the palygorskite in the two samples. At angle 2θ = 8.3°, an intense reflection of the palygorskite plane (110) is observed. The presence of quartz is confirmed by reflection at 2θ = 26.6°, with higher relative intensity in the raw palygorskite sample, in agreement with the mineralogical analysis. Dolomite carbonate is identified at angles 2θ = 30.9° and 41.1° in raw palygorskite; a peak referring to calcite is observed at 2θ = 29.4° in the Pharmasorb^®^ colloidal diffractogram (Figure 1).

LOI was conducted to assess the extent of weight reduction upon subjecting a sample to high temperatures in a muffle furnace. The LOI results, expressed as a percentage of the original sample weight (Table 1), revealed comparable values for both palygorskite samples. However, the raw palygorskite sample displayed a slightly higher percentage (14.38%) than the Pharmasorb^®^ colloidal sample (11.82%). This weight loss can be attributed to the volatilization of organic constituents, thermal decomposition of specific compounds (organic matter present in the material), or dehydration of crystalline hydrates [44].

### 3.2. Inductively Coupled Plasma Optical Emission Spectroscopy Analysis (ICP-OES) for Heavy Metals and Arsenic

ICP-OES analysis identified heavy metals as cadmium, cobalt, chromium, lead, nickel, and arsenic. Notably, the quantities of all elements detected comply with the specifications outlined in the British Pharmacopoeia [27]. It is worth mentioning that mercury was not detected in the analysis.

### 3.3. Methylene Blue Adsorption (MBA)

Furthermore, the findings regarding the adsorption capacity utilizing methylene blue as a model revealed that Pharmasorb^®^ colloidal exhibited a superiority of approximately 15-fold over raw palygorskite. This significant disparity suggests the need for further investigation into potential strategies aimed at modifying the structure of the silicate, with the objective of enhancing its molecular adsorption capacity.

### 3.4. Loss on Drying

The water molecules adsorbed on the surface and part of the zeolitic water inserted in the palygorskite tunnels are easily removed under low temperatures (<110 °C) [45]. Therefore, the loss by desiccation test showed losses lower than the limit stipulated by the British Pharmacopoeia (<17%), where raw palygorskite presented a loss of 6.36% and Pharmasorb^®^ colloidal 8.65%. This result is in agreement with the data from the thermogravimetric analysis. It is assumed that the slightly higher moisture adsorption in the commercial sample refers to the higher specific surface.

### 3.5. Acidity and Alkalinity

The pH values obtained for the samples are shown in Table 2. It is possible to observe that the aqueous suspensions have a basic character, given the high content of basic salts (dolomite and calcite) associated with their geological formation. Pharmasorb^®^ colloidal complied with pharmacopeial requirements (pH 7.0–9.5); however, raw palygorskite missed the upper margin slightly. According to Souza et al., impurities related to mineral carbonates can be easily removed after a simple acid treatment [46].

### 3.6. Surface Properties and Particle Size Analysis

Surface properties and granulometry (Table 2) are relevant aspects for the characterization of solid-state inputs, as they influence properties of pharmaceutical interest, such as adsorptive capacity and rheological profile.

Raw palygorskite has a BET-specific area of 122 m^2^/g, corresponding to approximately 60% of the measurement observed for Pharmasorb^®^ colloidal. The granulometric analysis of the palygorskite samples indicated an average micrometric size, with bimodal distribution due to the fibrous morphology of the material. The average diameter of raw palygorskite was 16.71 µm, while Pharmasorb^®^ colloidal had a smaller average diameter (5.5 µm). The particles have a heterogeneous distribution, with Span values of 3.56 and 3.48 for raw palygorskite and Pharmasorb^®^ colloidal, respectively. Both samples, when dispersed in distilled water, presented similar zeta potential values, thus confirming the negative character of the surface of the palygorskite fibers.

One of the most striking properties of fibrous clay minerals is their high specific surface area, attributed to their small particle size, the presence of channels and tunnels, and the morphology of the crystal [14]. Raw palygorskite presented a specific area of 122 m^2^/g, a similar value to those reported for samples from the same region: 113 m^2^/g [39], 118 m^2^/g [34], and 125 m^2^/g [47]. The superior specific area of Pharmasorb^®^ colloidal can be ascribed to the thermal treatment to which it is subjected. This thermal treatment can cause partial unobstruction of the channels and tunnels of the clay mineral.

Bearing in mind that palygorskite is mainly used as an adsorbent, tablet, capsule disintegrant, and tablet binder in solid pharmaceutical dosage forms, this property is of great relevance. Furthermore, the high specific surface area enables the adsorption of drugs as demonstrated by several authors [11,18,48,49].

The zeta potential is a measure used to characterize the electrokinetic potential of colloidal dispersions, whose value is influenced not only by the properties of the dispersed phase, but also by the pH, the ionic strength, and other parameters of the dispersing medium [50]. The surface can change its charge under the influence of the pH of the medium. This is due to the externally arranged Si-OH groups. In alkaline environments, the silanol groups are deprotonated, making the clay surface more electronegative, as demonstrated by Yang et al. [51]. However, a previous analysis conducted on the palygorskite extracted from the same geographical region determined the point of zero charge to be around pH 8 [40]. As a result, the surface of palygorskite has the potential to undergo protonation within a significant range of physiological pH conditions. This finding is particularly relevant when considering the material’s potential application for oral administration.

In addition, the zeta potential can help to predict the system’s stability. The surface charge can even correlate with palygorskite biocompatibility: some studies have reported that negatively charged particles have better biological compatibility, as they are not so easily internalized by cells due to electrostatic repulsion between the negative surface of the particles and the anionic glycosaminoglycans anchored in the cell membrane [52]. All these properties, as well as their similarities with Pharmasorb^®^ colloidal, indicate that raw palygorskite can be used as a pharmaceutical excipient. 

A parameter capable of influencing the specific area of fibrous phyllosilicates refers to the size of the fibers. Palygorskite is recognized as a 1D nanomaterial since the single crystal has a width of 20–30 nm and its length is generally of the order of micrometers, depending on its origin [53]. 

Both samples comply with the granulometric specification of the American Pharmacopoeia, which determines that palygorskite particles must have a particle size below 45 µm. The raw palygorskite sample has a larger mean diameter than Pharmasorb^®^ colloidal due to the industrial processing applied to this sample to reduce the size of its particles and break down the agglomerated fibers. The heterogeneity observed in the measurements is related to the random formation of these agglomerates, in which the crystals are not aligned or even parallel. The amplitude indicated by Span can also be attributed to the presence of contaminants, such as quartz, which correspond to 10–13% of the sample.

The results corroborate the difference found in the specific area of the samples since this parameter is inversely proportional to the size of the particles. The surface charges measured on palygorskite samples are similar. They are in line with data in the literature, which show a moderately negative charge due to the replacement of Al^3+^ by Fe^2+^ in the octahedral sheet [13,54,55,56]. 

### 3.7. Fourier Transform Infrared Spectroscopy

**Figure 2:**
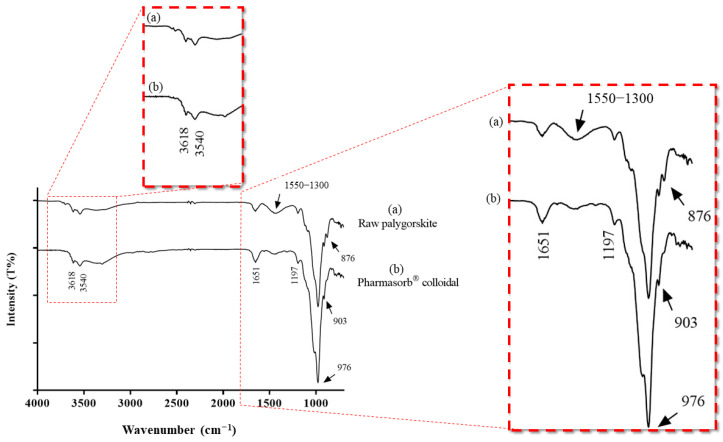
FTIR spectra of raw palygorskite (**a**) and Pharmasorb^®^ colloidal (**b**).

FTIR spectra curves show the similarity between the raw palygorskite and Pharmasorb^®^ colloidal (Figure 2). The spectrum in the infrared region of palygorskite is characterized by presenting signals referring to hydroxyl vibrations above 3000 cm^−1^, related to water, or around 1650 cm^−1^, referring to water in tunnels and channels. The bands of fibrous phyllosilicates appear below 1200 cm^−1^.

The bands related to structural hydroxyl stretching (Al-Al-OH) appear at 3618 cm^−1^, while the stretching of the hydroxyl corresponding to the coordinated and adsorbed water molecules appears at 3540 cm^−1^. The band at 1651 cm^−1^ belongs to the vibrational deformation of physiosorbed water and to the water molecules within the phyllosilicate channels. The band at 1197 cm^−1^ is characteristic of the sepiolite–palygorskite group due to tetrahedral inversion (Si-O-Si). The Si-O stretching vibration at 976 cm^−1^ and the Al-Al-OH deformation band at 912 cm^−1^ were properly identified at 976 cm^−1^ and 912 cm^−1^, respectively. The aforementioned bands coincide with reports in the literature [57,58,59].

In the raw palygorskite spectrum, an antisymmetric carbonate stretching band was observed between 1550 and 1300 cm^−1^, and a less intense band at 876 cm^−1^, typical of the carbonate anion, was also identified. Both have been previously described for palygorskite samples [60]; however, they are less intense in the Pharmasorb^®^ colloidal spectrum. This agrees with the XRF and XRD results, which indicate the presence of a higher number of carbonates in raw palygorskite with respect to Pharmasorb^®^ colloidal.

The band around 1600 cm^−1^ also has lower intensity in Pharmasorb^®^ colloidal than in raw palygorskite. This difference can also be ascribed to the thermal treatment of Pharmasorb^®^, which can remove the adsorbed water, the water contained in the channels, and even the coordinated water, depending on the temperature and heating time [61]. 

### 3.8. Thermal Analysis

**Figure 3:**
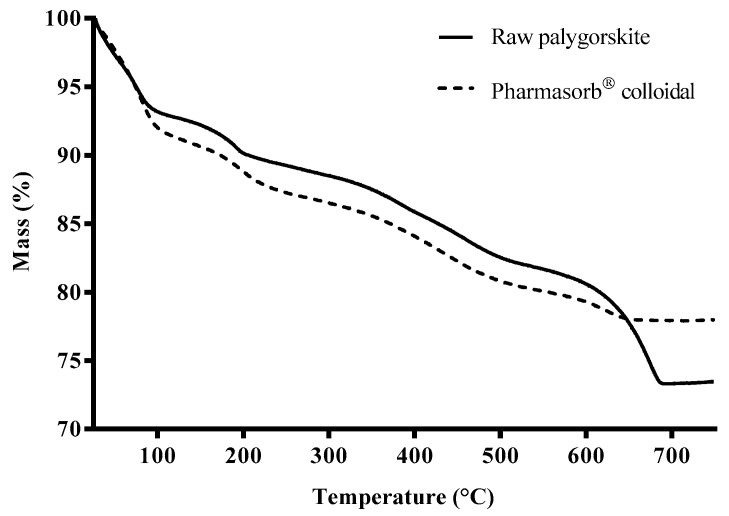
TG curves of palygorskite samples.

The thermogravimetric analysis of the samples demonstrated agreement with the literature, which describes the mass loss of this phyllosilicate in four steps (Figure 3 and Table 3). The succession of events can be divided as follows: the first event represents the loss of water adsorbed onto its surface, the second loss corresponds to the removal of water contained in the fiber channels, the third stage refers to coordinated water loss, and, finally, the dehydroxylation of the octahedral sheet is observed [62].

**Figure 4:**
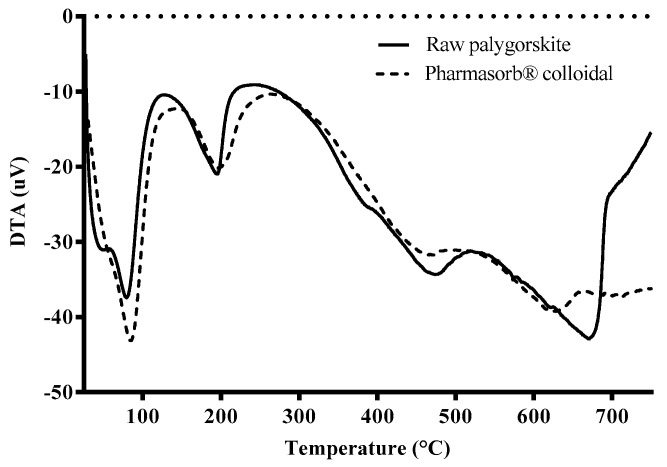
DTA curves of raw palygorskite and Pharmasorb^®^ colloidal.

The DTA curves (Figure 4) indicate a succession of endothermic events corresponding to the four stages of mass loss previously observed in the thermogravimetric analysis. Initially, it is observed that the raw palygorskite sample presents the first event divided into two stages, possibly related to moisture weakly adsorbed onto the surface of the phyllosilicate, given that it has not undergone any previous treatment, unlike the commercial clay, Pharmasorb^®^ colloidal. In the raw palygorskite sample, at temperatures below 100 °C, it is observed that the first event has a poorly resolved peak, which is related to surface adsorbed water. The second event refers to a partial loss of water in the phyllosilicate channels, as Rhouta et al., (2013) reported when analyzing Moroccan palygorskite samples [63]. The loss of residual zeolite water at 200 °C is similar to the two analyzed phyllosilicates. The loss of part of the water coordinated to the cations of the octahedral sheet of raw palygorskite was evidenced by a shoulder around 400 °C, while this event is less intense in Pharmasorb^®^ colloidal. The analysis of Chinese palygorskite samples reported by Cheng et al., (2011) demonstrated that the amount and type of impurity present influence the thermal behavior of phyllosilicates, such as calcite or dolomite [64].

A greater difference between the samples was found in the last thermal event, which was more intense and rougher for the raw palygorskite, probably due to the presence of dolomite. According to Cheng et al., (2011), the dehydroxylation of the clay mineral is followed by the decomposition of carbonates between 600 and 700 °C [64]. Therefore, the higher carbonate content in the crude sample justifies this loss [64]. The presence of dolomite in raw palygorskite justifies the higher mass loss of this sample above 600 °C compared to Pharmasorb^®^ colloidal.

The last event contemplates the condensation of the remaining structural hydroxyls, which require higher temperatures to diffuse due to the collapse of the channels, followed by the thermal decomposition of the carbonates. This loss was represented by an intense and extended endothermic peak in raw palygorskite (T_peak_ = 683.74 °C), justified by the significant presence of dolomite in the sample. A less sharp peak represented calcite decomposition in the DTA curve (T_peak_ = 627.53 °C) of Pharmasorb^®^ colloidal, corroborating the more subtle loss of mass of this carbonate in the thermogravimetric curve.

### 3.9. Electron Microscopy Analysis

**Figure 5:**
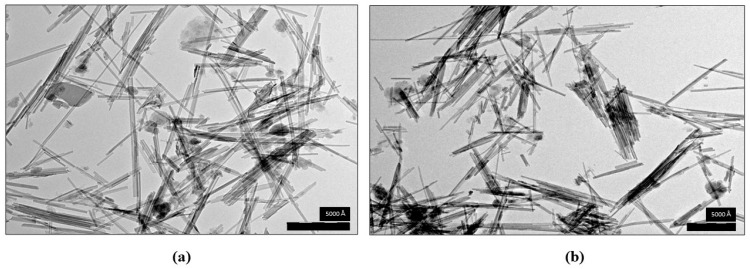
Transmission electron micrographs showing the size and morphology of raw palygorskite (**a**) from Piauí and Pharmasorb^®^ colloidal (**b**).

Analysis by transmission electron microscopy showed the acicular morphology of this phyllosilicate in both samples (Figure 5). Moreover, their macroscopic similarities are undeniable.

The crystals shown in Figure 5 occur as thin, elongated fibers of varying lengths, randomly distributed in different ways: isolated, grouped in a few units parallel to each other, or forming massive bundles from the agglomerated structures [65,66]. Pharmasorb^®^ colloidal fibers were shorter than in the raw palygorskite, which corroborates the data shown in Table 2.

At this point of the study, it seems clear that the main differences between raw palygorskite and Pharmasorb^®^ colloidal mainly lie in the amount of secondary mineral phases. Although the amount of impurities is low compared with most phyllosilicate deposits, a further purification of raw palygorskite could easily increase its quality and potential uses. 

Purification operations, such as the removal of coarser quartz particles and acid leaching to reduce carbonates and iron oxides, are quite straightforward. Such steps would increase the surface area of the raw palygorskite and minimize possible incompatibilities between the phyllosilicate and drugs susceptible to oxidation, thus exerting a significant impact on the final uses of the Brazilian palygorskite.

Raw palygorskite has shown several positive attributes that make it a viable choice for use in pharmaceutical and cosmetic production. It meets safety and quality requirements established by the British Pharmacopoeia, is more cost-effective compared to Pharmasorb colloidal, is readily available, and benefits from a well-established value chain. 

Palygorskite is a biocompatible raw material that has been extensively studied for its safety in vitro assays involving various cell types. These studies have demonstrated its biocompatibility with macrophages, fibroblasts, renal epithelium, and human cervical cancer cells [6,67,68]. Furthermore, palygorskite is recognized as an adjunct in the treatment of diarrhea and is also listed as a pharmaceutical excipient in non-parenteral formulations in American and European pharmacopeias. In a recent publication by our group, the biocompatibility results of microparticles prepared with chitosan and the same raw palygorskite were presented. The study employed BALB/3T3 clone A31 embryonic fibroblasts from mice, and the results demonstrated high biocompatibility, indicating satisfactory outcomes [69]. These factors, along with its proven efficacy in the pharmaceutical industry, indicate its quality and safety for use in such applications.

## 4. Conclusions

Historically, clay minerals have played an important role in human history, from their uses in the building industry to healthcare, and especially in the pharmaceutical field. In fact, they are widely used as excipients and active ingredients themselves, appearing in medical and pharmaceutical textbooks. However, to be used in the pharmaceutical field, they must meet minimum quality and purity standards. This manuscript aims to characterize a raw palygorskite clay mineral extracted from a Brazilian deposit and compares it with a pharmaceutical-grade palygorskite commercialized as Pharmasorb^®^ colloidal. To this end, both samples were characterized using X-ray fluorescence, X-ray powder diffraction, BET surface method, granulometry, zeta potential, infrared spectroscopy, thermal analysis, and electron microscopy. 

The results confirm that the clay mineral extracted from the Brazilian deposit can be considered a palygorskite, this being the main mineral phase present. The secondary mineral phases found were also equivalent for both samples. Moreover, the physicochemical properties of the Brazilian sample, even without previous treatments or purification, were very similar to those of Pharmasorb^®^ colloidal. Due to its high specific surface area, particle size, and moderated negative charge. The exploitation of such a high quality, natural resource of palygorskite for the healthcare field can be of benefit to not only the healthcare field itself but it could also foster the growth and development of Brazil. This becomes even more important when we consider that the number of deposits of such high purity in this mineral is low. Brazilian raw palygorskite meets all the minimum quality standards that enable it to be exploited from a pharmaceutical perspective, and for it to potentially be applied in non-parenteral drugs and cosmetics.

## Figures and Tables

**Table 1:** Chemical composition of palygorskite samples.

Oxides (%, *w*/*w*)	Raw Palygorskite	Pharmasorb^®^ Colloidal
SiO_2_	53.70	59.57
MgO	10.68	8.94
Al_2_O_3_	10.25	10.22
CaO	4.63	3.83
Fe_2_O_3_	4.09	3.08
K_2_O	1.00	0.91
TiO_2_	0.90	0.70
MnO	0.27	0.05
P_2_O_5_	0.05	0.82
Na_2_O	0.05	0.06
Loss on ignition	14.38	11.82

**Table 2:** Surface and granulometric properties of raw palygorskite and Pharmasorb^®^ colloidal.

Sample	Specific Surface Area (m^2^/g)	pH *	Zeta Potential (mV) *	Particle Size Distribution (µm)			
				d10	d50	d90	Span
Raw palygorskite	122	9.63 ± 0.10	−13.1 ± 0.9	1.40	11.38	41.96	3.56
Pharmasorb^®^	196	8.76 ± 0.06	−15.5 ± 0.1	1.14	3.43	13.09	3.48

* Data were represented as mean ± standard deviation (*n* = 3).

**Table 3:** Thermal events related to phyllosilicates obtained from TG and DTA curves.

Sample	Temperature Range (°C)	T_peak_ (°C)	Weight Loss (%)	Residue (%)
Raw palygorskite	30–88.90	87.92	5.72	74.01
	88.90–200.58	211.97	3.57	
	200.58–485.41	478.37	7.19	
	485.41–686.07	683.74	9.51	
Pharmasorb^®^ colloidal	30–96.27	84.77	7.13	78.73
	96.27–219.25	197.26	4.27	
	221.25–489.62	472.37	6.93	
	489.62–644.64	627.53	2.94	

## Data Availability

Not applicable.
